# Golgi-Targeting Anticancer Natural Products

**DOI:** 10.3390/cancers15072086

**Published:** 2023-03-31

**Authors:** Myat Nyein Khine, Kaori Sakurai

**Affiliations:** Department of Biotechnology and Life Science, Graduate School of Engineering, Tokyo University of Agriculture and Technology, Tokyo 184-8588, Japan

**Keywords:** natural products, anticancer agents, Golgi apparatus, stress response, cancer signaling

## Abstract

**Simple Summary:**

A selected set of natural products that target the structure and function of the Golgi apparatus in cancer cells is highlighted in this review. The potential for modulating these Golgi-mediated signaling pathways for anticancer drug development is also discussed.

**Abstract:**

The Golgi apparatus plays an important role in maintaining cell homeostasis by serving as a biosynthetic center for glycans, lipids and post-translationally modified proteins and as a sorting center for vesicular transport of proteins to specific destinations. Moreover, it provides a signaling hub that facilitates not only membrane trafficking processes but also cellular response pathways to various types of stresses. Altered signaling at the Golgi apparatus has emerged as a key regulator of tumor growth and survival. Among the small molecules that can specifically perturb or modulate Golgi proteins and organization, natural products with anticancer property have been identified as powerful chemical probes in deciphering Golgi-related pathways and, in particular, recently described Golgi stress response pathways. In this review, we highlight a set of Golgi-targeting natural products that enabled the characterization of the Golgi-mediated signaling events leading to cancer cell death and discuss the potential for selectively exploiting these pathways for the development of novel chemotherapeutic agents.

## 1. Introduction

The Golgi apparatus in vertebrates comprises stapled stacks of flattened membrane cisternae, which are fused together to form a characteristic ribbon structure. It is a key organelle in the secretory pathway responsible for the transport of proteins, sterols and lipids to their target sites [1]. Consequently, it plays a crucial role in keeping different cellular organelles distinct and functioning properly. The Golgi apparatus is also where the vital cellular components, glycans and lipids are synthesized and proteins are modified. The biosynthetic enzymes such as glycosyltransferases display distinct localization across subcompartments of the Golgi apparatus to coordinate a precise sequence of enzymatic reactions in an orderly fashion [2,3]. In addition to these canonical functions, the Golgi apparatus is now recognized as a signaling hub for mediating various cellular processes such as cell growth, mitosis, apoptosis, autophagy, ferroptosis and stress responses [4,5,6,7]. It has been demonstrated that altered signaling events within the Golgi apparatus contribute to the development of various diseases, including cancer [8,9,10,11]. These cancer-adapted signaling processes can be viewed as a vulnerability of cancer cells that can be exploited for development of selective anticancer drugs.

**Figure 1:**
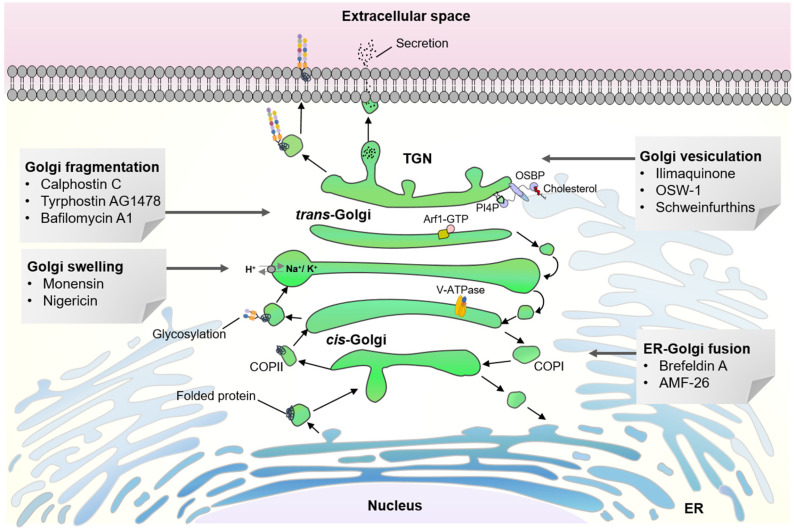
Structural organization of mammalian Golgi apparatus is important for vesicular transport, biosynthesis of glycans and lipids, post-translational modification of proteins and intracellular signaling.

**Figure 2:**
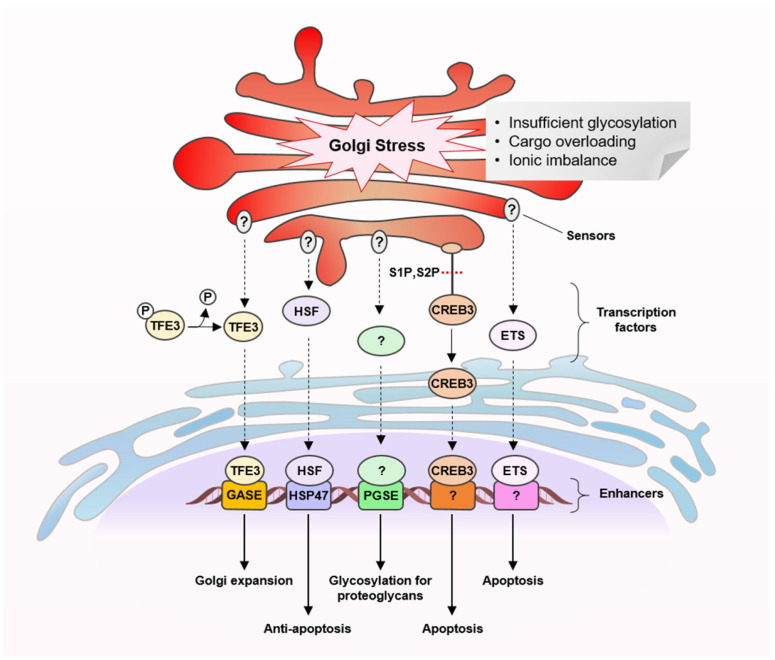
Golgi stress response pathways.

Bioactive small molecules that can specifically perturb or modulate Golgi proteins and organization have been instrumental in the structure and functional studies of the Golgi apparatus (Figure 1) [12,13,14,15]. They allowed dissection of complex and dynamic cellular signaling pathways by targeting specific proteins in a spatiotemporal manner. A number of natural products with anticancer property have been identified as powerful chemical probes in deciphering Golgi-related pathways and, in particular, recently described Golgi stress response pathways (Figure 2) [16,17]. Considerable advancement has been made in the understanding of the role of organelles in the mechanisms of cellular response to various stresses in order to maintain homeostasis [17]. A well-characterized example can be found in the ER stress response pathway [18,19,20]. More recently, specific responses to stresses imposed on the Golgi apparatus have attracted attention as potentially promising drug targets for treating cancer, in which abnormal survival and migration increase the demands on Golgi function [16,17]. Natural products continue to represent a major source of candidate molecules for drug discovery and development [21,22,23]. Therefore, anticancer agents that specifically localize to the Golgi apparatus may provide attractive platforms for development of novel Golgi-targeting chemotherapeutic agents [24,25].

**Figure 3:**
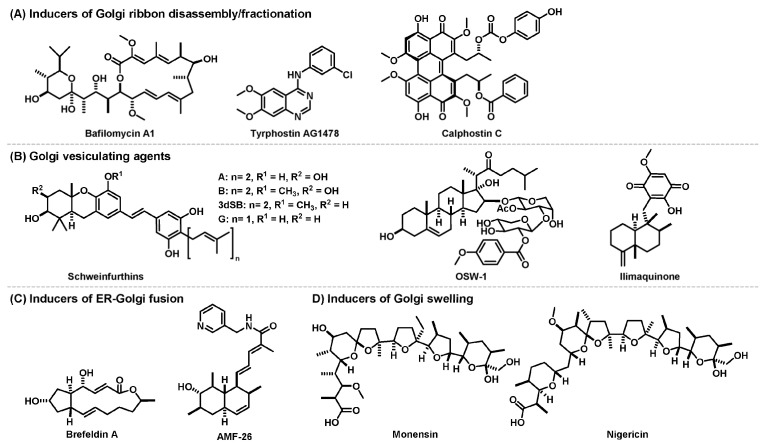
Anticancer natural products that disrupt structural organization of Golgi apparatus.

In this review, we summarize current knowledge on the Golgi stress response pathways (Figure 2) and highlight natural products and their derivatives on the basis of the following biological activities: anticancer activity and their ability to target the Golgi apparatus by disrupting their structural organization and function (Figure 3). We also discuss possible links between their known anticancer mechanisms and Golgi stress responses and the potential utility of the Golgi-targeting natural products in harnessing cancer-adapted processes for therapeutic application.

## 2. Golgi Stress Response Pathways

In order to fulfill cellular demands, the functional capacity of different organelles in a cell is tightly regulated by a process named organelle autoregulation [17]. The Golgi stress response has newly emerged as one of these homeostatic mechanisms, similar to the more well-established ER stress responses [16,17,18,19,20]. In response to various perturbations incurred in the Golgi apparatus, its capacity is enhanced to supply the cellular needs, for instance, an increase in the vesicular flux, by upregulating post-translationally modified proteins and transportation of secretory and membrane proteins (Figure 2). To ensure proper functioning of the Golgi apparatus, its structural organization is dynamically regulated [1,26,27]. Although much is still not well understood, uncontrolled alteration of the Golgi structure, for example, ribbon disassembly or fragmentation, can lead to disruption in the secretory pathway and a signal that triggers stress responses. When the Golgi function is severely compromised by stress-inducing events beyond repair, some of the response pathways eventually lead to cell death [16].

To date, several types of molecular pathways for Golgi stress response have been described, transcription factor E3 (TFE3), cyclic AMP-responsive element-binding protein 3 (CREB3), heat shock protein 47 (HSP47), proteoglycan (PG) and mitogen-activated protein kinase MAPK-ETS pathways (Figure 2) [16,17,28,29,30,31,32,33]. Similar to ER stress response pathways, these pathways are proposed to comprise a set of sensor molecules, a transcription factor, a transcriptional enhancer element and target genes encoding Golgi-related proteins. Each of the Golgi stress response pathways appears to respond to different triggers, although many of the molecular details are yet to be clarified.

The TFE3 pathway promotes the expression of *N*-glycosylation enzymes (SIAT4A, SIAT10, FUT1, B3GAT2 and UAP1L1), Golgi structural proteins (GCP60, GM130 and Giantin) and vesicular transporter components (STX3A, WIPI49 and RAB20) by activating transcription factor TFE3 that binds and activates an enhancer element, the Golgi apparatus response elements (GASEs). It has been shown to respond to Golgi-disrupting agents such as monensin and nigericin (Figure 3) but the sensors of the pathway are unidentified [16,28,29]. Several Golgi-disrupting agents such as brefeldin A (BFA) and AMF-26 (Figure 3) can induce cell death via the CREB3 pathway, in which ARF4 has been identified as a target gene [30]. ARF4 is a member of the small G protein family that recruits coat proteins and adapter proteins and helps promote cargo transport from the Golgi apparatus. Downregulation of the transcription factor CREB3 has been shown to mimic the loss of ARF4. In the HSP47 pathway, which can be triggered by benzyl α-GalNAc, an *O*-glycosylation inhibitor, an unknown factor activates expression of the ER chaperone HSP47 to prevent Golgi stress-induced apoptosis [31]. The PG pathway is activated when glycosylation of proteoglycans becomes insufficient, for example, by treatment of 4-nitrophenyl-β-d-xyloside, a β1,4-galactosyltransferase inhibitor. It upregulates transcription of genes involved in the glycosylation of Golgi proteoglycans including those of glycosyltransferases, an isomerase and sulfotransferases [32]. Two types of PG-type Golgi stress response element (PGSE), termed PGSE-A and PGSE-B, have been shown to regulate this pathway, while sensors and transcription factors are yet to be identified. The MAPK-ETS pathway is mediated by the ETS family of transcription factors, ELK1, ETS1 and GABPA/B [33]. Golgi stressors such as BFA, monensin (Figure 3) and golgicide A were shown to activate MEK1/2 and extracellular signal-regulated kinase (ERK) 1/2, indicating that the MAPK signaling cascade activates ETS transcription factors to promote cell death.

## 3. Golgi Stress-Inducing Anticancer Natural Products

Given the fact that the Golgi apparatus plays a central role in the secretory pathway, changes in Golgi structure and function are expected to affect the homeostasis of post-translationally modified proteins, glycans and lipids [1,5,6,9]. Various cellular responses, such as inflammation, and many diseases and disorders, from neurodegeneration to cancer, are associated with the loss of Golgi ribbons and the appearance of a dispersed Golgi apparatus [8,9,10,11,34,35]. The structural organization of the Golgi apparatus is inherently dynamic and undergoes structural transformation during the cell cycle and under stress conditions through tubule formation, tubular stack assembly and disassembly, fragmentation and vesiculation [26,27]. Under normal cell growth conditions, this transition is highly regulated and the vesiculation of Golgi ribbons is called Golgi haze at the onset of mitosis, which are reorganized into a new set of ribbons assembled at the mitosis exit. The molecular mechanisms of how different stress triggers cause different types of structural disruption to the Golgi apparatus are currently not well understood. The continual maintenance of an ordered structure is vital to the survival of cancer cells and therefore the key components facilitating the Golgi reorganization can be considered attractive targets for effective anticancer agents. In addition, elucidation of direct target proteins and the mechanism of Golgi structural disruption for the Golgi-targeting natural products should enable rational design of more potent analogs and more precise modulation of Golgi-related signaling for treatment of cancer.

In this section, we categorize Golgi-disruptive anticancer natural products according to their effects on the structural organization of the Golgi apparatus (Figure 3). Different patterns of structural disruption can be characterized with the target substructures and the extent of the impact of disruption [5,6]. They may be indicative of the specific way the Golgi apparatus responds to a certain type of disruption. Accordingly, we differentiate typical phenotypes into four groups: ribbon disassembly, vesiculation, fusion with ER, and swelling. We provide a summary of natural products that induce the respective phenotypes and signaling processes that are involved in these events as well as their possible consequences.

### 3.1. Inducers of Golgi Ribbon Disassembly/Fragmentation

#### 3.1.1. Calphostin C

Calphostin C was isolated as the most potent compound among the calphostin family of perylenequinones from the fungus *Cladosporium cladosporioides* in an inhibitor screen for protein kinase C (PKC) [36,37]. It selectively inhibits PKC over PKA with the IC_50_ value of 0.05 μM and >50 μM, respectively [38]. This activity is light-dependent, and is correlated to its antiproliferative activity against human tumor cells HeLa S3 (GI_50_ = 0.23 μM) and MCF-7 (GI_50_ = 0.18 μM) [36,37]. Calphostin C triggers apoptosis via caspase-3, caspase-9, and caspase-7 in mouse NIH 3T3 cells and human MCF-7 cells [39,40]. PKC isozymes are key regulators of intracellular signaling pathways, which are associated with cell proliferation, migration, tumorigenesis and metastasis [41,42]. Overexpression of PKC, therefore, has been recognized as a significant anticancer drug target. Calphostin C has received considerable attention as a promising drug lead due to its unique properties [43,44]: it generates singlet oxygen upon photoirradiation, which would make it a suitable photosensitizer for photodynamic therapy, and it selectively binds to the diacylglycerol (DAG)/phorbol ester site present in the C1 regulatory domain of PKC [36,37,38], unlike other non-selective kinase inhibitors such as staurosporine that bind to the catalytic domain [45].

Through a light-dependent mechanism, calphostin C has been shown in NRK cells and HT-29 cells to cause fragmentation and dispersal of Golgi stacks into clusters of vesicles and short tubes, similar to the residual Golgi structures present in normal mitotic cells [46,47]. It also inhibited both exocytic and endocytic vesicular transport. However, calphostin C-induced Golgi disassembly was not due to PKC inhibition but was proposed to involve direct binding to a structural Golgi membrane protein having a phorbol ester-binding domain. This disassembly process also required protein phosphorylation. In MCF-7, PANC-1 and U251 cells, calphostin C at 50 nM, in similar concentration ranges to its GI_50_ values, induced extensive vacuolation of the ER, which was correlated with an activation of ER stress response and inhibition of ER-to-Golgi trafficking of glycoproteins [40].

#### 3.1.2. Bafilomycin A1

Bafilomycin A1 is a macrolide antibiotic derived from *Streptomyces griseus* that inhibits vacuolar-type H^+^-ATPases (V-ATPases) with high potency and selectivity [12,48,49,50]. V-ATPases are ATP-driven proton pumps responsible for acidification of intracellular compartments such as lysosomes and in certain cell types, extracellular space. They modulate enzyme activities and protein sorting that are important for membrane trafficking, autophagy and apoptosis [51]. A recent cryo-EM study revealed that bafilomycin A1 binds to two c subunits of bovine V-ATPase, and consequently prevents proton conductance [52]. Its ability to inhibit V-ATPases, which is known to be important for tumor cell invasion and migration, makes it a potential anticancer agent [51,53,54]. It has also been widely used as an endosomal neutralization agent and autophagy inhibitor [12,55].

Bafilomycin A1 has been reported to inhibit V-ATPases on the Golgi membrane, which acidifies the Golgi lumen to a pH of 6.5, leading to neutralization of Golgi compartments [56]. Although the general morphology of the Golgi apparatus was largely unaffected, bafilomycin A1 treatment accumulated long tubules at the *cis*-face of the Golgi apparatus while decreasing the number of p58-positive COPI vesicles in Hep-2 and HeLa cells. These observations suggested that the drug inhibits protein retrieval from the post-ER zone. Neutralization of Golgi apparatus by bafilomycin A1 also resulted in relocalization of glycosyltransferases in HeLa and LS174T cells, which generated glycoproteins with shortened *O*-glycans [57]. Bafilomycin A1 induces caspase-independent cell death in hepatocellular carcinoma cells via targeting autophagy and the MAPK pathway, which is related to one of the recently emerged Golgi stress response pathways [16,58]. As an inhibitor of the secretion of the proteins from the *trans*-Golgi network (TGN), bafilomycin A1 was used to study the interaction of HSP47, a Golgi stress response-related protein, and procollagen through the secretory pathway [31,59]. HSP47 was shown to dissociate between the post-ER and the *cis*-Golgi compartments and it suggested that its C-terminal RDEL sequence may serve as an ER retention signal.

#### 3.1.3. Tyrphostin AG-1478

Tyrphostin AG-1478 (AG-1478) is a synthetic quinazoline analog of the natural product erbstatin and is a highly active and specific EGFR tyrosine kinase inhibitor with an IC_50_ of 3 nM [60]. AG-1478 serves as an ATP-competitive inhibitor of the kinase domain. As a close analog of erlotinib, which is an approved drug for non-small cell lung cancer patients with EGFR mutations [61], AG-1478 has attracted great interest as a potential anticancer EGFR inhibitor that can be used to treat cancers with overexpressed EGFR, including non-small cell lung, pancreatic, breast and colorectal cancer, and was moved into a clinical trial for the treatment of glioblastoma multiforme (GBM) with constitutively active EGFR overexpression [62,63,64]. The cytotoxic effect of AG-1478 is much higher than that observed solely by EGFR inhibition, suggesting that the compound elicits its activity both via EGFR-dependent and EGFR-independent pathways. AG-1478 was also found as a hit compound in an image-based screen for inducers of Golgi disassembly [65,66]. It was subsequently found to inhibit the Sec7 domain of GBF1, a guanine nucleotide exchange factor for ARF. The E794K mutation in GBF1 causes complete disassembly of the Golgi apparatus, in a similar manner to that in BFA- and AG-1478-treated cells. Thus, it was suggested that the inhibition of GBF1 function by AM-1478 phenocopies the catalytically inactive E794K GBF1 mutant. While having a similar GBF1 inhibitory function to that of BFA, AG-1478 displays lower cytotoxicity and a more selective effect toward *cis*-Golgi proteins with no endosome tubulation, unlike BFA. AG-1478 was found as a hit compound with IC_50_ = 1 μM in yet another high-content imaging screen for inhibitors of the trafficking of α-mannosidase II (Man II) from the ER to the Golgi apparatus. It was shown in HeLa cells to disperse Golgi protein markers, GM130, TGN46, GalT and giantin, which was partially reversible [67]. Since erlotinib and other related tyrphostins did not affect the transport of Man II nor disrupt Golgi structure, it was proposed that the effect of AG-1478 on Golgi structure and function is independent of its EGFR inhibitory activity.

### 3.2. Golgi-Vesiculating Agents

#### 3.2.1. Ilimaquinone

Ilimaquinone (IQ) is a major sesquiterpene metabolite of several *dictyoceratida* sponges, which displays anti-HIV, anti-inflammatory and antimitotic activities [12,68]. IQ reversibly inhibits vesicular transport of newly synthesized proteins from the ER to the medial Golgi membranes, causing a dramatic vesiculation of the Golgi apparatus throughout the cytoplasm [69]. IQ-induced Golgi vesiculation has been shown to require sequential reactions of phospholipase D (PLD) and phosphatidic acid (PA) phosphatase that generates DAG from PA [68]. It was also found that the DAG production by IQ also promotes subsequent PKD activation, which is considered to mediate Golgi fragmentation [70,71].

Photoaffinity labeling and affinity chromatography studies identified one of the methylating enzymes, *S*-adenosylhomocysteinase (SAH), as a specific target protein for IQ. IQ inhibits SAH activity, suggesting that IQ perturbs cellular methylation events [72]. Protein and lipid methylation are important for vesicular transport and inhibitors of SAH are known to block vesicle-mediated secretion in a similar manner to IQ. IQ displays antiproliferative activity in several cancer cell lines, including prostate cancer PC-3 and LNCaP, non-small cell lung cancer A549 and hepatocellular carcinoma Hep3B, with GI_50_ values ranging from 2.6 μM to 12 μM, while that of normal human umbilical vascular endothelial cells (HUVECs) was shown to be much higher (GI_50_ > 30 μM) [73]. It was suggested that its antiproliferative activity is induced via G1 arrest with increased expression and nuclear translocation of CHOP/GADD153 in PC-3 cells. IQ has been shown to induce apoptosis in HCT-116 cells (GI_50_ = 17.89 μM) via mitochondrial pathway as evidenced by DNA fragmentation, loss of the mitochondrial membrane potential, increased gene expression levels of caspase-9 and caspase-3 and decreased Bcl-2 gene expression [74]. Whether the Golgi vesiculation by IQ is directly linked to the anticancer activity of the compound remains to be established.

#### 3.2.2. OSW-1

OSW-1 is a steroidal saponin isolated from bulbs of the African lily *Ornithogalum saundersiae* that displays extremely potent antiproliferative activity against various cancer cell lines, with an average GI_50_ = 0.78 nM in the National Cancer Institute (NCI) 60 human tumor cell line screening [75,76]. Significantly, it exhibited as much as a 100-fold selective cytotoxicity against cancer cell lines over non-malignant cells. Its structure is unique among the saponin class of natural products due to the acylated disaccharide moiety at the D-ring of the steroidal aglycone moiety. As the bioinformatics analysis using the COMPARE program predicted a novel mechanism for OSW-1 that is distinct from those for known anticancer drugs, it has attracted great interest as a new candidate compound for development of selective anticancer agents [77,78,79,80]. However, the molecular mechanism of its selective anticancer activity has not been fully elucidated.

The target proteins of OSW-1 have been reported to be OSBP and ORP4L [81,82], which are members of the OSBP-related (ORP) family involved in sterol/lipid sensing and transport [83,84]. OSBP primarily resides in the cytosol and translocates to TGN upon sensing cholesterol depletion or binding to oxysterol such as 25-hydroxycholesterol [83,84,85]. It facilitates cholesterol/PI4P exchange at the membrane contact site between the ER and TGN [86,87]. OSW-1 was shown to preferentially localize to the Golgi apparatus [88] and induces Golgi fragmentation, presumably via OSBP binding [81,88]. On the other hand, ORP4L depletion was shown to induce dispersal of the Golgi-localized TGN46 and GALNT2, suggesting that ORP4L might also be involved in regulating TGN and *cis/medial*-Golgi morphology [89].

It was demonstrated that OSW-1 selectively induces Golgi stress response pathways over ER stress response by activating TFE3, inducing transcriptional activation of pro-apoptotic ARF4 as well as transcriptional downregulation of antiapoptotic HSP47 [90]. While depletion of OSBP has been known to result in Golgi fragmentation [83,90], it was also found to activate TFE3, which appears to be phenocopied by OSW-1 treatment [90]. However, OSBP knockdown is not lethal and does not explain the OSW-1-mediated apoptosis. Although OSW-1-induced cytotoxicity needs to be explored, its ability to selectively induce Golgi stress response raised the possibility to develop Golgi-targeting anticancer agents.

#### 3.2.3. Schweinfurthins

The schweinfurthins are geranyl- or prenylstilbenes found from the leaves and fruits of *Macaranga* plants, as well as from propolis produced by bees harvesting from them [91,92,93]. They characteristically exhibit potent and differential activity in human tumor cell lines, including those cancer types that had been traditionally difficult to treat such as glioblastoma multiforme (GBM) and triple-negative breast cancer (TNBC). Schweinfurthin A and B are the most potent among the family compounds, with the mean GI_50_ of 0.36 μM and 0.81 μM, respectively, in the NCI 60 human tumor cell line assay [91]. COMPARE analysis of their cytotoxicity profiles identified no other compounds of known mechanism of action, but found strong correlations with cephalostatins, OSW-1 and stelletins, which were all predicted to act through novel target(s) and mechanism(s) [81,91,92].

While the differential cytotoxicity of schweinfurthins is still poorly understood, it was in part ascribed to their ability to disrupt the cholesterol homeostasis pathway [94,95,96,97,98]. For example, a synthetic analog, 3-deoxyschweinfurthin B (3dSB), upregulates LXR target genes, such as ABCA1, ABCG1 and LDLR, which leads to increased cholesterol efflux and decreased cholesterol uptake. Interestingly, an in vitro binding assay showed that it does not directly bind LXRα nor LXRβ. This analog was also shown to trigger the phosphorylation of eIF2α and the increased expression of GRP78 and PDI, leading to ER protein-folding deficiency and ER stress response [96]. Like OSW-1, schweinfurthin A has been reported to target OSBP with nanomolar affinity and to a lesser extent ORP4L [81]. Schweinfurthin A induces translocation and accumulation of OSBP to TGN, although gross structural alteration of this site was not observed unlike in the case of OSW-1 treatment [81,91]. Similarly, schweinfurthin G was shown to bind OSBP and inhibit its sterol/PI4P exchange activity in vitro at a nanomolar concentration range [97]. Prolonged treatments with schweinfurthin G were shown to cause TGN disruption and to arrest TGN trafficking [97,98]. These conditions, furthermore, result in altered glycosylation of cell surface proteins, downregulation of PI3K/mTOR/Akt signaling and induction of ER stress response [98].

### 3.3. Inducers of ER–Golgi Fusion

#### 3.3.1. Brefeldin A

Brefeldin A (BFA) is a lactone antibiotic found in *Penicillium brefeldianum*, *Angelica sinensis* and *Curvularia lunata* that inhibits vesicular traffic of many animal cells [99]. BFA triggers a rapid and reversible vesiculation of *cis*-Golgi network and TGN. It causes both the formation of uncoated membrane tubules, through which Golgi components redistribute into the ER, and the failure to transport molecules out of this fused ER–Golgi structure. The target protein of BFA has been identified to be GBF1, which mediates the forming of transport vesicles by recruiting COPI coat proteins to cargo-bound receptor proteins present on the Golgi membrane [100]. As a classic example of inhibitors of protein–protein interaction (PPI), namely, ARF1–ARFGEF interaction, BFA has played a crucial role in the investigation of vesicular traffic. The molecular basis of Golgi disruption by BFA has been extensively studied. It inhibits the initial interaction of ARF1 with the membrane, leading to an impaired formation of transport vesicles, affecting the transport of proteins and lipids to different intracellular destinations [101]. It has been widely used as a chemical probe in inducing both ER and Golgi stress response pathways [14,102,103].

BFA was found to induce cancer cell death by triggering autophagy, in which upregulation of binding immunoglobulin protein Bip is induced, inhibiting the phosphorylation of Akt and mTOR [104]. The structure–activity relationship studies of BFA and its derivatives have suggested that the rigid ring structure, the α,β-unsaturated lactone and 4-hydroxyl groups are necessary for its ability to induce DNA fragmentation and apoptosis in HCT-116 [16,105]. Because BFA displays low selectivity for cancer cells and poor physical and pharmacokinetic properties, its clinical use has been hampered. Nevertheless, it has provided an attractive lead compound to develop an anticancer agent and considerable efforts have been directed toward synthesis of BFA analogs and derivatives with improved pharmacokinetic properties and reduced cytotoxicity [106].

#### 3.3.2. AMF-26

AMF-26, or M-COPA, is a trichodermic acid derivative, found as a hit compound from an antitumor activity screening against the JFCR39 human cancer cell line panel in a search for new anticancer drug candidates having a similarity index to BFA using the COMPARE analysis [106]. Although the structure of AMF-26 is distinct from BFA, it displayed potent antitumor activity with a similar mean GI_50_ value to that of BFA of 47 nM (mean GI_50_ = 43 nM) and appeared to share a similar Golgi-disrupting mechanism involving inhibition of the ARF1–ARFGEF interaction. The BFA-sensitive large ARFGEFs, GBF1, BIG1 and BIG2, which are *cis*-Golgi network and TGN resident proteins, activate ARF1 to recruit COPI and facilitate Golgi–ER vesicular transport [107]. Depletion of GBF1, BIG1 and BIG2 by siRNA has been shown to induce Golgi dispersion and cell growth inhibition, which is phenocopied by AMF-26 treatment. In neoplastic mast cells and gastrointestinal stromal tumors (GISTs), an inhibition of protein trafficking from ER to Golgi by AMF-26 resulted in suppression of Kit autophosphorylation, which led to cell growth inhibition [108,109].

AMF-26 was also identified as an angiogenesis inhibitor, which inhibits the phosphorylation of VEGF receptor 1 (VEGFR1), VEGFR2, Akt and ERK1/2 stimulated by VEGF in HUVECs [110]. Oral administration of AMF-26 markedly inhibited angiogenesis induced by VEGF or IL-1β. It was shown that AMF-26 exhibited anticancer effect by inhibiting transport of receptor tyrosine kinases RTKs to the cell surface [107,111]. In tyrosine kinase inhibitors (TKIs)-resistant small cell lung cancer cell lines, AMF-26 induced downregulation of MET, which is also known as hepatocyte growth factor receptor, suggesting that anticancer mechanism of AMF-26 was different from that of known EGFR-TKIs. In preclinical studies, AMF-26 was demonstrated to be effective against HER2-negative, MET-positive gastrointestinal tumors that are characterized by gene amplification of MET by downregulating its membrane expression [110].

### 3.4. Inducers of Golgi Swelling

#### 3.4.1. Monensin

Monensin is a highly selective sodium ionophore polyether antibiotic, produced by *Streptomyces cinnamonensis*, that causes characteristic swelling of Golgi cisternae [12,112]. It folds into a 1:1 complex with a cation such as Na^+^ and it can readily partition into biomembranes in the neutral, protonated carboxylic acid form, then releases Na^+^ ions in the bulk solution, thus serving as a Na^+^/H^+^ exchanger. It is the first ionophoric antibiotic approved for use by the FDA for animal use and has been known as a food additive to cattle and chicken feed to promote growth by enhancing intestinal absorption of calcium and sodium ions [113]. Monensin represents one of the classic protein secretion blockers. Both monensin and BFA are used as inhibitors of extracellular protein transport, although their mechanisms of action are thought to be different [12,113]. It has been employed in a large number of studies on Golgi structure including recent reports that described the TFE3-mediated Golgi stress response pathway [14]. The molecular detail of monensin’s action on the Golgi apparatus is still not well understood. It is believed to cause osmotic swelling of *cis*- and *trans*-Golgi networks, thereby preventing protein secretion from the luminal face of the cisterna to the outside, by disrupting the acidification mechanism at the Golgi membrane via Na^+^/H^+^ antiport [12,113]. In cell models as well as in the cellular environment, monensin displays preferential binding to the cholesterol-rich membranes, including that of the Golgi apparatus. It was suggested that the more pronounced effect of monensin on the Golgi apparatus than those on lysosomes or other organelles may be explained by the fact that the *trans*-Golgi membrane has a higher cholesterol level than the others except for the plasma membrane [10]. Moreover, swollen vesicles produced by monensin treatment were found to be enriched with cholesterol [12]. As the neutralization of Golgi lumen globally affects various pH-controlled processes in subcompartments of this organelle, monensin inhibits biosynthetic enzymes and, in particular, glycosylation enzymes.

Monensin has been reported to suppress many cancer-related pathways such as those involving β-catenin/Wnt, E2F/DP1, STAT1/2, NFkB, AP-1 and Elk-1/SRF, and to downregulate EGFR expression, especially in pancreatic cancer lines [114,115]. Monensin inhibits colon cancer cell growth by inducing cell cycle arrest and apoptosis [116]. Therefore, it has been suggested as a potential candidate for repurposing for cancer treatment [115,117].

#### 3.4.2. Nigericin

Nigericin is a polyether antibiotic derived from *Streptomyces hygroscopicus*, which was first isolated in 1950 [118]. Based on its similar structure to monensin, it displays a cation-binding ionophore property similar to that of monensin. Interestingly, it acts as a selective antiporter of K^+^/H^+^ rather than as a Na^+^/H^+^ antiporter and raises the pH of acidic compartments. Like monensin, nigericin treatment was reported to induce reversible *trans*-Golgi swelling in HeLa cells as observed by stably expressing GalT-GFP as a *trans*-Golgi marker [119] Thus, nigericin appears to neutralize the acidic Golgi apparatus and causes Golgi expansion, which as a consequence, interferes with Golgi functions such as glycosylation and vesicle trafficking. The acidifying effect of nigericin in cytosol results in inhibition of DNA synthesis in cancer cells [120]. Nigericin was also reported to promote the maturation and release of IL-1β from lipopolysaccharide (LPS)-stimulated cells by decreasing intracellular levels of K^+^, which is required for IL-1β activation [121]. In colon cancer cells (SW620 and KM12), it was shown to suppress the Wnt/β-catenin signaling pathway and induce apoptosis at low μM concentrations. It suggested that nigericin treatment enhanced the degradation of β-catenin, which was mediated by the β-catenin destruction complex involving GSK-3β/Axin 1. Although the molecular basis of nigericin-induced downregulation of β-catenin is still not clear, in particular with respect to the nigericin’s ionophore activity, the finding raised the possibility that nigericin may be useful for colon cancer treatment.

## 4. Conclusions

Golgi stress response pathways are emerging as potentially promising yet complex targets for cancer prevention due to the dynamic nature of the organelle and involvement of multiple proteins. Since the Golgi apparatus plays an essential physiological role in mammalian cell homeostasis, selective modulation of target components in the stress response pathway is required for development of selective anticancer drugs and chemical probes to further study the yet to be characterized mechanisms underlying this pathway.

Similar to those natural products that target ER stress response pathways, many Golgi-targeting natural products are discovered through phenotypic screening and have been mostly used as useful chemical tools to manipulate or study Golgi stress response-related signaling. Perhaps as a consequence, Golgi-targeting agents have not been actively considered as suitable lead compounds for anticancer drugs because of their known cytotoxic effects or pleiotropic effects. However, more recently, there have been several reports that point to the therapeutic potential of Golgi-targeting anticancer agents and increasing efforts are directed toward synthetic development of natural product analogs with cancer-selective activity. For example, AMF-26 has entered clinical studies for targeting lung cancer with resistance toward KRAS inhibitors. In parallel, such selective agents are advancing our understanding on the complex molecular mechanism of Golgi-mediated processes. With the availability of protein structures, in silico design and screening methods, target engagement analysis methods, comprehensive metabolomics methods and HTP screening assays, the discovery of new lead compounds or development of more selective agents should be more feasible.

The greatest advantage that natural products offer is the privileged scaffold structures that nature has selected over many years of evolution that display specific activity intercepting the signaling network for supporting cellular homeostasis. The examples summarized in this review underscore the uniqueness of Golgi-targeting natural products having distinct scaffold structures and modes of action, in comparison with currently available anticancer drugs. With a rise in the interest in medium-sized drugs, as well as development of various selective drug delivery strategies, it is anticipated that complex natural products that modulate Golgi function should be taken into consideration as selective anticancer drug candidates.
